# Rescue and Translocation of Hispaniola Hutia (*Plagiodontia aedium*) in Pueblo Viejo, Cotuí Mining Concession Area

**DOI:** 10.1002/ece3.70560

**Published:** 2024-11-13

**Authors:** Miguel S. Núñez‐Novas, Rosanna Carreras‐De León, Amelia L. Mateo Jiménez, Carolina Dávila, Pilar Calderón

**Affiliations:** ^1^ Natalus Consultoría Ambiental S.R.L. Santo Domingo Dominican Republic; ^2^ Barrick Pueblo Viejo Dominicana Jersey 2 Limited Pueblo Viejo Dominican Republic

**Keywords:** conservation, hutia, industry, Rodentia, translocation

## Abstract

Translocation of species is a common practice used in endangered species management and conservation plans, which can have the purpose of establishing new populations which are at risk of being wiped out, increase a species chance of survival or recovery, and to preserve genetic diversity. The present study details the rescue and relocation process of Hispaniola Hutia (*Plagiodontia aedium*) in the municipality of Cotuí, Dominican Republic. Data are presented for eight individuals, belonging to two different families, detailing capture methods, handling, reintroduction method, and behavior, as well as data on post‐release monitoring for both families. It was found that the most efficient capture method was active capture by hand (6/8). The family groups were heterogeneous, although both families had in common the presence of a single adult male. The average weight of the individuals was 1.19 kg. Blood tests were carried out on the animals before being taken to the holding pens and compared with the values of other rodents, showing that hematic values for *P. aedium* were like those of another hutia species, *Cynomys ludovicianus*. During the period animals were held in pens before reintroduction, hutias showed trophic preferences for *Clusia rosea* and *Guarea guidonea*. The only supplementary food they consumed was *Daucus carota*. A fight was observed between members of different families during the acclimation period in the holding pen, between a subadult male and another dominant one, evidencing territorial behavior. Post‐release monitoring of the families showed that the selection of habitat for relocation, based on the characterization of active burrows in the area, was successful in one of the two families relocated. One of the families abandoned the selected area for translocation during the monitoring. It is important to note that accurate population data of the targeted population, such as abundance and density, are necessary to guarantee the survival of the translocated animals in the new environment. The reintroduction method used in this study may serve as a baseline for future translocation projects for the species.

## Graphical abstract

Data are presented for eight individuals, belonging to two different families, detailing capture methods, handling, quarantine period, and behavior, as well as data on post‐release monitoring of the two families. Clinical and hematological data of the individuals have been included. We also present a habitat selection matrix that will help as a basis to apply in future relocations for the species, if additional data on the population density for the area to be relocated are included.

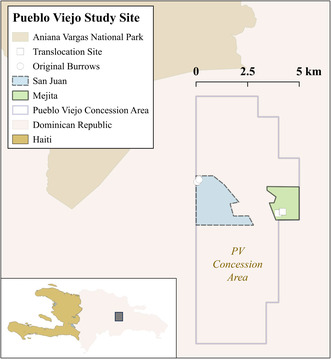

## 1 Introduction

Translocation involves the movement by humans of living organisms from one part of their range to another (Griffith et al. 1989; IUCN 2013b). In general, these wildlife movements are usually beneficial to humanity, although they can lead to major environmental problems, such as in the cases of *Urva auropunctata*, *Lithobates catesbeianus*, and *Rhinella marina*, the introduced exotic species which affected local biodiversity (Finkle 2012; IUCN 2013a; Simons, Lee, and Haney 2013). Translocations are usually stressful for individuals, due to the lack of knowledge that they have of their new environment (Aguilar‐Cucurachi et al. 2010; Bosson, Palme, and Boonstra 2013). Because of this, IUCN drafted in 1987 a Position Statement on Translocation of Living Organisms (IUCN 1987). Later, the Species Survival Commission's Reintroduction Specialist Group developed a set of guidelines approved by the IUCN council in 1995 and published in 1998 as Guidelines for Reintroductions and Other Conservation Translocations (IUCN 2014). In the Dominican Republic, the need to translocate wildlife for conservation has increased due to urban expansion, agriculture, mining, and touristic development.

### 1.1 Hispaniolan Hutia

Hispaniolan hutia is an endemic rodent species with restricted distribution across the island and is listed as Least Concern by the IUCN Red List of Threatened Species and the National Red List of Threatened Species from the Dominican Republic (Kennerley and Turvey 2024; MIMARENA 2018). The social structure of Hispaniolan hutia is like other hutia species from Cuba (Silva Taboada, Duque, and Franco 2007), where individuals are organized in independent family groups with one dominant adult male, subordinate males, and one or two adult females with their offsprings. Solitary males and females have been observed for *Capromys pilorides* in Cuba (Berovides 1998). *Plagiodontia aedium* and *Solenodon paradoxus* (Solenodon) have been recognized globally as conservation priority species (Collen et al. 2011), being the last remaining terrestrial mammals on the island, shared politically by the Dominican Republic and Haiti.

Recent efforts have been made to understand the species ecology and spatial distribution on the island, developing potential distribution maps based on historical data, the collection of sightings from naturalists and biologists, and habitat modeling using current climatic parameters (Brace et al. 2012; Kennerley, Nicoll, Young, et al. 2019; Turvey et al. 2020). However, population studies for both species throughout their distribution range and characterization of the usage of vegetation have not been studied.

**FIGURE 1:**
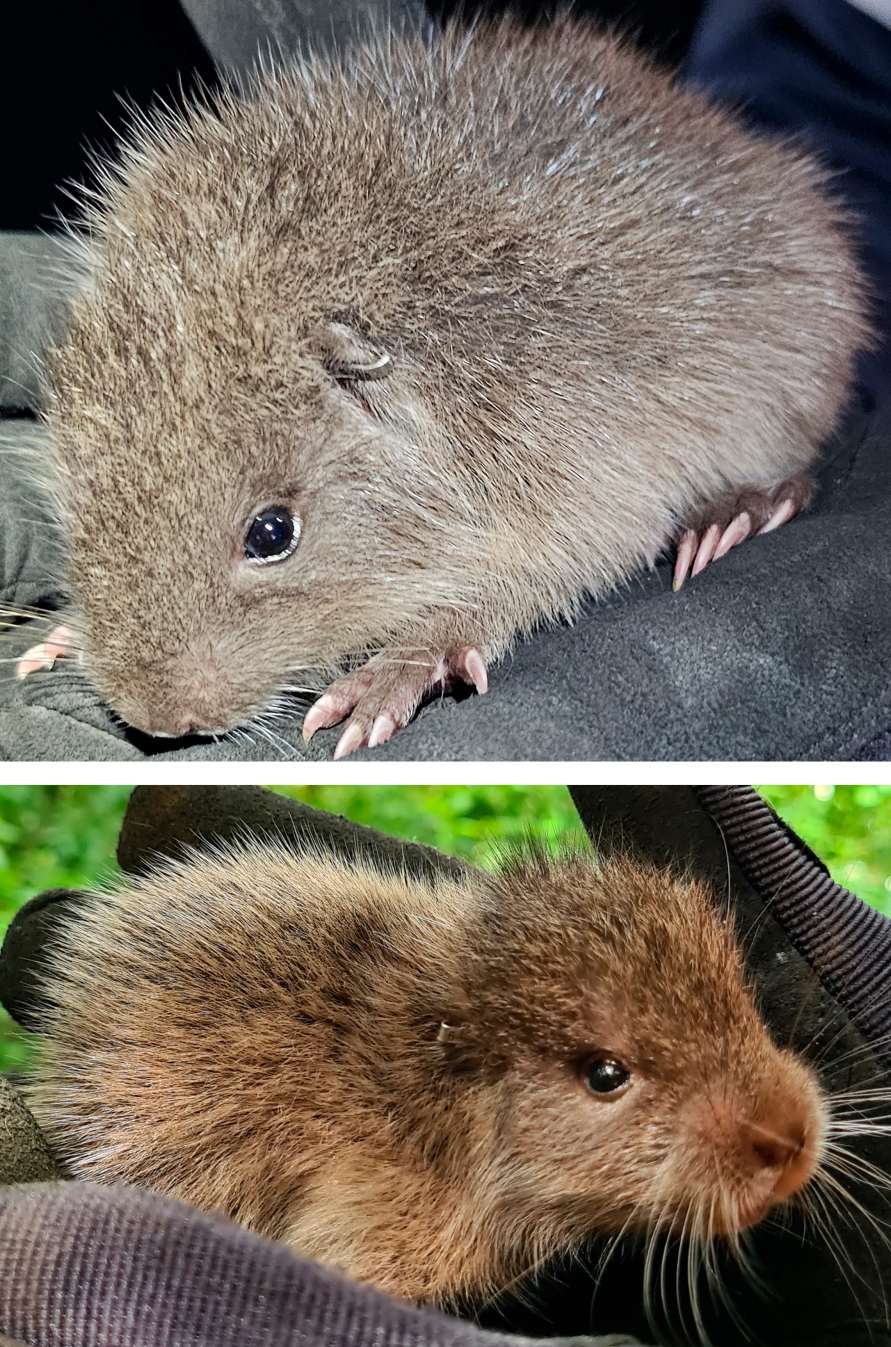
Juvenile from Family 1 of *Plagiodontia aedium* from the mining concession area in the Dominican Republic.

Hispaniola hutia (*Plagiodontia aedium* Cuvier 1836; Figure 1) is an understudied polytypic species, consisting of three subspecies, *P. aedium aedium*, *P. aedium hyaleum*, and the recently described *P. aedium bondi* (Turvey et al. 2015). Most studies involving *Plagiodontia* genus have addressed taxonomic aspects, with limited publications regarding its natural history and ecology (Kennerley, Nicoll, Young, et al. 2019; Woods and Ottenwalder 1992). More studies on population abundance and density need to be done to better understand the species' population trend over time and to advise local conservation efforts.

In this study, we present results from a rescue and translocation plan taken place within the mining concession area, Barrick Pueblo Viejo—PV, located in the Dominican Republic. Karstic rock is mined from the concession area for its use in the raw material processing for the extraction of silver and gold. Due to the exploitation of karstic rock, the main habitat for *P. aedium*, the need to translocate animals has become the most feasible conservation measure within this area. The presence of this endangered species in the operational area represents a risk for the individuals belonging to that population, hence their rescue and translocation is urgently needed. Morphometric data, physiological parameters, and clinical evaluation during this study for eight specimens belonging to two different families of hutia are included.

## 2 Materials and Methods

### 2.1 Study Area

**FIGURE 2:**
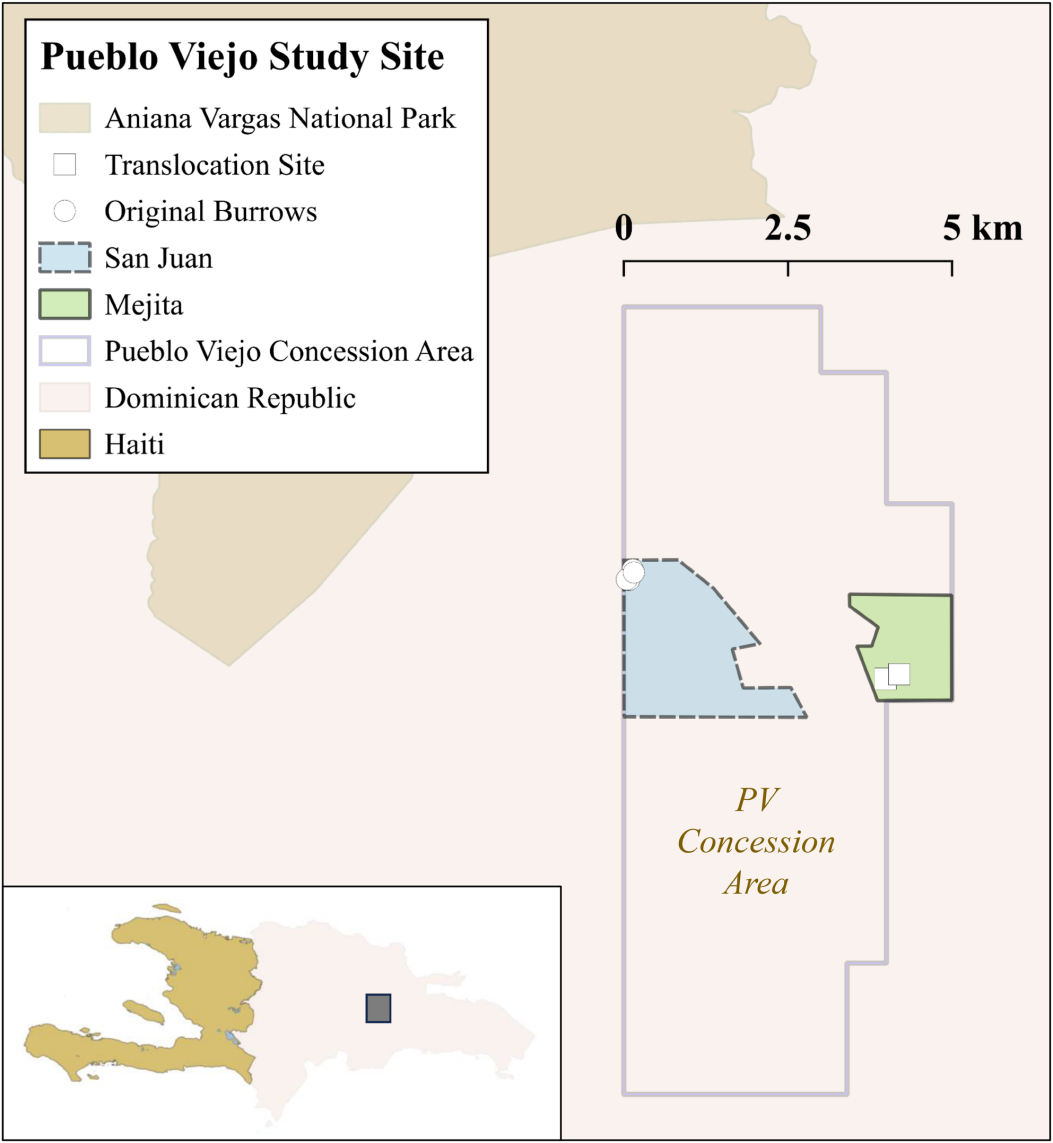
Study area. Pueblo Viejo (PV) mining concession area in the Dominican Republic. Hutia original burrows in white circles and potential burrows in squares.

This study was carried out in the Pueblo Viejo mining concession area located within the municipality of Cotuí, province Sánchez Ramírez in the Dominican Republic (Figure 2). This area is mined for gold and silver by Barrick *Pueblo Viejo Dominicana Jersey 2 Limited* company (Barrick Pueblo Viejo—PV). PV is in the northeast of the Cordillera Central Mountain system, with Hatillo Dam and the National Park Aniana Vargas at its northwest. The study area within Pueblo Viejo includes two sections referred to as Mejita and San Juan Hill (Figure 2). Within the study area, Mejita section was selected to translocate the animals given that it holds the highest density of individuals and the highest number of refuges and foraging habitats. Therefore, this site was targeted as the optimal site to relocate the hutia families from the vulnerable San Juan site. Despite there being other potential translocation sites near the concession area, such as Aniana Vargas National Park (Figure 2), the decision to translocate in the nearest optimal site was influenced by multiple factors such as governmental permit authorization. Mejita population is under the protection and surveillance of the Environmental Department of PV, making post‐monitoring most feasible. Also, given that this was the first translocation experiment for PV, it was decided that it was best to translocate the animals in the site we had more information from.

### 2.2 Translocation Protocol

All translocations were carried out in compliance with IUCN protocols (Guidelines for Reintroductions and Other Conservation Translocations; IUCN 2014) and adapted to the site and species requirements. This included animal rescue from San Juan site, animal handling and transportation to holding pen for acclimation, and reintroduction to new habitat. The translocation method included the population restoration practice of *reinforcement* as the main activity which is defined as the “intentional movement and release of an organism into an existing population of conspecifics” (IUCN 2014, 2). IUCN (2014) guidelines specify that these types of translocations should have a long‐term commitment plan for their monitoring to guarantee their successful establishment in the translocated site.

### 2.3 Habitat Selection

#### 2.3.1 Habitat Characterization

Habitat evaluation was conducted from February 23 to March 1 of 2022 within the PV concession area. The main objectives were to identify, characterize, and describe active burrows within the land targeted for exploitation and search and characterize potential burrows (PB) in the habitat selected for translocation. Active burrows were detected in previous exploration trips by expert trackers covering all known occupancy areas of hutia within the concession site. For burrow detection, first trackers detected the foraging sites, which are regularly in close proximities to active burrows. Once an active burrow and/or potential burrow for reintroduction was identified, several parameters for their description were taken: coordinates (UTM‐WGS84), altitude (masl, meters above sea level), number of entrances, predominant substrate, canopy cover (densitometer), plant species associated to the burrow, species of plants used by hutias surrounding the burrow, directionality of the entrance and slope, and soil temperature, and a qualitative description of the burrows was determined with a snake camera (OiiWAK‐10M). The vegetation associated to the active hutia sites in Mejita and San Juan hill was evaluated with two methods, transect methodology and the placement of circular parcels in the detected active and potential burrows. A total of eight transects were done, six in Mejita and two within the San Juan hill. Data on the type of forest, key species present, and plant species with a specific use by hutia were recorded, such as leaf gnaw, gnawed trunk (green or mature), scratches on trunks, gnawed on exposed roots, and the presence of feces. The transect length varies between 100 and 150 m and 2 m wide. The associated vegetation to active and potential burrows was described by establishing a circular parcel with a 10 m radius around each burrow included. Circular parcels were placed in the center of the active burrows, taking the entrance of the burrow as the central point of the parcel. We identified all plant species within the circular parcels.

#### 2.3.2 Selection of Potential Sites Through Ranking Matrix

With the data gathered from active burrows within the concession area, a scoring system was designed to classify potential burrows' relative quality. The criteria selected are listed in Table S1. These criteria were used to establish a range of values which describe some basic parameters to determine the optimal environmental and physical characteristics for a hutia burrow. Altitude ranges are the ones observed within the concession area, and soil temperatures are based on temperatures obtained during the dry season (January and February 2022). Criteria No. 10 is based on a preliminary list of 24 species of common plants identified in active burrows across the optimal habitat within the concession area (Table S2). These data were based on the data obtained from the circular parcels described in the next section.

The potential burrows (PB) within Mejita section in the concession area were searched by expert hutia trackers from the Dominican Republic, and once they identified potential burrows, parameters for their characterization were taken and compared with the parameters taken for active burrows. Additionally, camera traps were installed in the potential burrows for 3 days with the objective of verifying the availability of the burrow for a new family and the presence of potential threats present, such as invasive species (cats, dogs, and mongoose) and human activity. Only burrows with minimal threat from invasives were considered. Two potential burrows were preselected for this study based on their quality, which we determined with a quality ranking matrix design specifically for this site and species (PB‐04 and PB‐05; Figure 2).

### 2.4 Sampling and Animal Processing

#### 2.4.1 Capture

Capture of individuals from San Juan hill was done by hand and using Tomahawk Traps (dimensions: 76.2 cm length, 22.9 cm width; 22.9 cm height) when needed. The area of capture was delimited using the active burrow entrances as central points and expanding the search radius to 184 m (Kennerley, Nicoll, Young, et al. 2019) and during high activity hours (7:00 PM to 7:00 AM). Hutias were tracked using signs of their presence such as recent feces, recently gnawed trunks, and leaves. At night, trackers also used animal vocalizations to detect animals to capture. Once an individual was detected in the field, the trackers determined the best way to approach the animal to capture by hand using thick gloves and head lamps. Once animals were caught, they were introduced in breathable bags to transport to the processing site in Mejita.

#### 2.4.2 Animal Processing

All individuals were ear‐tagged with a 1 cm metal tag used for mice (Kent Scientific Corporation). Clinical evaluation and animal processing were conducted at the Mejita site. Clinical profile for all two families were done in two separate moments, post‐capture and pre‐release. A physical exam was performed and included the following: cardiac frequency, respiratory frequency, and body temperature; visual inspection of eyes, ears, oral cavity, skin, hair, and nails. Blood and fecal samples were taken, and ectoparasite specimens were also collected. To minimize stress due to animal manipulation, heart and respiratory rates were taken with the animal resting over a table in ventral decubitus position, inside a partially open cloth bag. The bag blocked vision, facilitating auscultation without the need for restraint. A neonatal Littmann classic II stethoscope was used; this model was used due to the small size of its bell, which allows listening to the heart and respiratory sounds of adult, juvenile, and neonate specimens. The bell of the stethoscope was placed between the 2nd and 3rd intercostal space, on the left sternal border, using a mobile stopwatch; the beats were heard for 15 s and counted, and the result was multiplied by 4 to obtain the total beats per minute. The respiratory frequency (RF) was obtained by visualizing the exhalations evidenced by the expansive thoracic movements carried out in a period of 15 s; later, the result was multiplied by 4 to obtain the total number of breaths per minute (R/min). Body temperature was measured using a digital thermometer that was introduced into the anal orifice after applying a water‐based lubricating gel. Eyes, ears, oral cavity, skin, hair, and nails were inspected with the naked eye, searching for pathological signs.

Each specimen was weighted using Pesola scales and sexed. Weight (kg) was taken in two separate moments, post‐capture and pre‐release for all individuals. Morphometric data were taken using a rigid extensible ruler in units of cm, as well as a digital caliper, where the measurements of the following structures were obtained: head–body length (H‐BL), tail length (T‐L), right and left ear length (E‐L), total length (Total L), and right and left hind leg length (HL‐L). Age class and reproductive stage were recorded for all individuals. Additional morphometrics on reproductive traits were obtained: for males, penis length (P‐L), anus–penis distance (AP‐D), penis base length (P‐BL); for females, clitoris length (C‐L), anus–vulva distance (AV‐D), clitoris base length (C‐BL), and vulva base length (V‐BL).

During containment, the individuals defecated, and with the help of a dissecting forceps, fecal samples were collected for three separate individuals, 1 g each. Fecal samples were weighed on a digital scale with a capacity of up to 1 kg. Subsequently, they were deposited in sterile bottles with screw caps and 100 mL rated capacity.

#### 2.4.3 Phlebotomy

For phlebotomies, 3 mL syringes and 23 G x 1 hypodermic needles were used. The puncture area was disinfected by spraying 75% isopropyl alcohol and drying with cotton. Phlebotomies were attempted in the lateral caudal vein of the tail. Also, venipuncture in the saphenous vein was obtained, managing to collect 3 mL of whole blood from an individual from family 1, which was distributed in two collection tubes, one with EDTA and the other without additives, respectively.

To study the hematic and biochemical profile of the specimens, all samples were processed in the National Bio Vet Laboratory (NBVL), in Miami, Florida, United States of America, a veterinary laboratory that offers the processing of samples with specific values for rodents. Tubes of 3 mL were used because this value is less than 10% of the blood volume of an adult individual and the minimum required by the laboratory to perform both tests. Biochemical profile analyses were sent through Ramvet lab that outsources to NBVL service laboratories in Florida, USA. The package included 25 tests: A/G Ratio, Albumin, ALK Phos, ALT (SGPT), Amylase, AST (SGOT), B/C Ratio, BUN, Calcium, Chloride, Cholesterol, CO_2_, CPK, Creatinine, Direct Bilirubin, Globulin, Glucose, GGTP, LDH, Osmolality, Phosphorus, Potassium, Sodium, Total Bilirubin, and Total Protein.

### 2.5 Soft‐Release Technique

**FIGURE 3:**
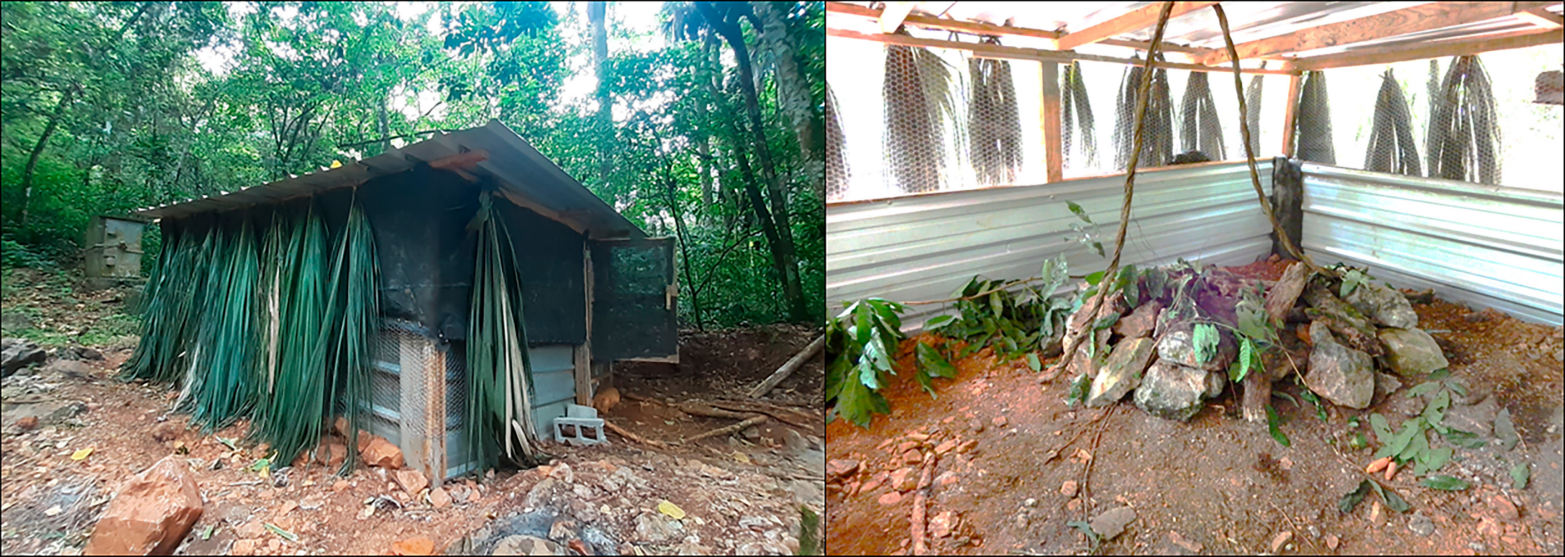
Left: Enclosure for acclimation period (corral) located in Mejita. Right: Burrow constructed within corral with vegetation added for foraging.

A soft‐release reintroduction technique involved translocating hutia individuals into a close enclosure of 9 m^2^ and 2 m of height located in Mejita natural habitat. The enclosure was fabricated with wood, metallic mesh, saran mesh, and aluminum roof panels (Figure 3). Additional shade was given with dry palm tree leaves. Within the enclosure, two burrows were constructed with cement blocks and a wooden box that serve as an artificial burrow (dimensions: 71.1 cm length, 45.7 cm width, and 33.0 cm height). Also, logs and tree branches from their natural habitat were used to give the burrow a vegetation cover. The cement blocks were organized in a single line to fabricate an exit tunnel. Enrichment of both the burrow and perching was done with natural vegetation obtained from their habitat. The acclimation period for each animal in the holding pen varied per individual, where the first individual captured lasted no more than 15 days in the enclosure (Table 2). The main purpose of applying the soft‐release technique was to offer some acclimation period and gather the entire family before reintroduction, given that not all members of each family were captured the same day. The temperature in Celsius (°C) and relative humidity for the enclosure were recorded daily during this time.

#### 2.5.1 Food Item Selection

Food item selection was done using data from the literature for Cuban hutia (Amaro‐Valdés et al. 2019) and data obtained from the vegetation surveys performed in this study. Two types of food item selection were done, food items present in their natural habitat and supplementary food items. The first type of food items consisted of leaves and branches of plant species from Table S2. Supplementary food item included fresh vegetables and fruits obtained from the local market, which included carrot, corn, batata, avocado, and others. Food items from the natural habitat were collected from Mejita area, close to the enclosure, and inserted into the corral. The frequency of food item offering varied between 1 and 3 days depending on the amount of foraging (gnawed leaves and trunks) on the placed food items.

#### 2.5.2 Behavior

Behavior and monitoring of individuals within the holding pen were recorded with camera traps (Infrared Reconyx). For each family, three camera traps were installed inside their section within the holding pen. To assure the welfare of the animals, the cameras were revised every other day for signs of stress on individuals, fights between males, female submission during reproductive stages, fights for food, attacks to juveniles, and other behaviors that may indicate a risk for animal welfare.

### 2.6 Monitoring Post‐Release

Once animals completed the acclimation period, they were reintroduced to Mejita into a preselected site based on the quality determined by the ranking matrix (PB‐04 and PB‐05; Figure 2). Release of the animals was done between 8 and 10 AM for individuals to enter their new burrow and explore during the day. Once animals were released, the burrow was not visited during the following 3–5 days to avoid affecting their adaptation process. Families were released 215 m apart from each other in Mejita. Two camera traps (Infrared Reconyx) were installed on each burrow before reintroduction, one in a direct line to the entrance of the burrow and a second camera in the predicted diagonal path of their movement in and out of the burrow. Monitoring with camera traps had a duration of 3 months, and camera batteries and data were collected twice a month.

## 3 Results

### 3.1 Sampling

**FIGURE 4:**
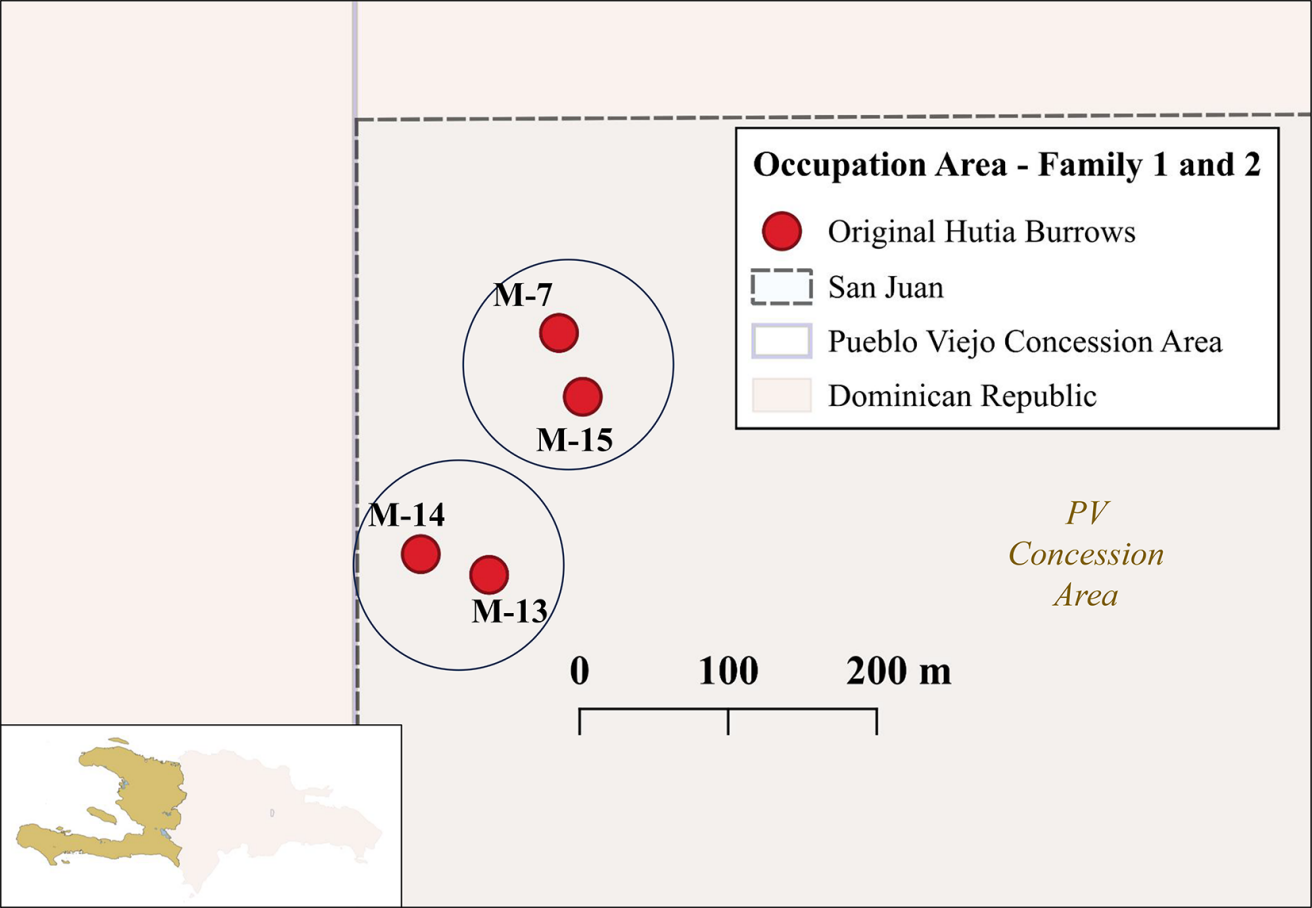
Occupation area for families 1 (M‐7 and M‐15) and 2 (M‐13 and M‐14) in San Juan Hill within the Pueblo Viejo (PV) mining concession area.

A total of eight individuals were captured in San Juan hill belonging to two different families. Both families occupied two different burrows within San Juan Hill (Family 1: M‐07 and M‐15; Family 2: M‐13 and M‐14) (Figure 4). Family 1 had a female with its offspring (Figure 1) along two other males. Female and offspring were captured on the same day, May 18, and placed in the holding pen together, while the last individual of Family 2 was captured on June 1. Family 2 presented two females and two males. Five out of the eight individuals captured were full‐grown adults (Table 1). Capture dates can be seen in Table 1.

**TABLE 1:** Captured hutia individuals' general data.

	Capture date	ID hutia	Ear tag right	Ear tag left	Sex	Age class
Family 1	May 18	Pa‐01	6475	6421	Male	Juvenile
	May 18	Pa‐02	6472	6471	Female	Adult
	May 19	Pa‐03	6470	6424	Male	Adult
	May 21	Pa‐04	6499	6500	Male	Subadult
Family 2	May 24	Pa‐05	6477	6476	Male	Adult
	May 25	Pa‐06	6423	6422	Female	Adult
	May 30	Pa‐07	6450	6448	Female	Adult
	June 1	Pa‐08	6457	6458	Male	Subadult

The most efficient capture method was by hand capture (6/8 success rate), in which the trackers had to be in the proximities of the burrow targeted prior to the animals leaving to forage. Once the hutia was identified visually or acoustically by the trackers, they proceeded to hand‐capture it. Hutias emit a vocalization like a screech, which becomes more acute when they are stressed. This screech is very particular and allows them to be located at night, when they are looking for food or moving (Kennerley, Nicoll, Butler, et al. 2019). The use of Tomahawk traps for capture was less efficient (2/8 success rate) due to the adaptation period that is necessary to wait for capture, and at the same time, there were no data on the most preferable baits to attract the Hispaniola hutias. This technique was only effective when the animals were forced to enter the cages by limiting the exits and placed directly in front of their main burrow exit. This latter technique is not recommended when little to no damage is intended to the hutia's burrow surroundings. The installation of the trap involved some habitat modification to reduce the escape area. Hence, it is not recommended when the habitat is intended to be preserved in the long term.

### 3.2 Habitat Characterization and Selection

Sixty plant species organized in 40 families were identified in the eight transects done (Table S4). Fourteen species showed gnawing and traces of hutia on their branches, bark, or leaves, as was the case of *Chrysophyllum cainito*. The vegetation in San Juan hill is regenerating from past agricultural practices (remanent cacao plantations), and the presence of old infrastructure material indicated the usage of that habitat by humans not too long ago.

In the Mejita zone, during the exploration of optimal habitat for reintroduction and potential burrows (PB), a total of six circular vegetation parcels were done. Out of these six parcels, two were determined to be better suited for hutia reintroduction (PB‐04; PB‐05) based on the habitat characterization ranking matrix (Table S1). A total of 15 and 18 different plant species were identified for PB‐04 and PB‐05, respectively (Table S3).

A total of 71 species in 44 families were identified in the transects and plots. About 40 species were unique to the transects, 10 were unique to the plots, while 19 were common between transects and plots. Of the 71 species, about 14 species were used in some way by hutias, of which three were found in transects and plots (Table S4).

### 3.3 Animal Processing

The average weight of the eight specimens was 1.19 kg (SD = 0.39). All individuals lost weight during their acclimation time within the enclosure. Individual Pa‐02 (female) lost the most weight (weight difference: −0.28 kg) spending 15 days in captivity prerelease. Pa‐08 showed the least amount of weight loss (−0.01 kg) and had spent the least amount of time in the corral (7 days). Average male adult weight was 1.40 kg (SD = 0.00), and female adult average weight was 1.43 kg (SD = 0.16). The average total length for males was 43.42 cm (SD = 7.08) and for females, it was 47.33 cm (SD = 2.31) (Table 2).

**TABLE 2:** Morphometric data of hutia individuals from San Juan Hill.

Family 1					Family 2				
	Weight					Weight			
	Weight post‐capture (kg)	Weight pre‐release (kg)	Weight difference	Days in corral		Weight post‐capture (kg)	Weight pre‐release (kg)	Weight difference	Days in corral
Pa‐01	0.40	0.32	−0.09	15	Pa‐05	1.40	1.23	−0.17	15
Pa‐02	1.41	1.14	−0.28	15	Pa‐06	1.60	1.42	−0.18	14
Pa‐03	1.40	1.28	−0.12	14	Pa‐07	1.27	1.18	−0.09	9
Pa‐04	1.21	1.14	−0.08	12	Pa‐08	0.83	0.82	−0.01	7

	Morphometrics					Morphometrics			
	HB‐L (cm)	T‐L (cm)	Total L (cm)	Age class‐sex		HB‐L (cm)	T‐L (cm)	Total L (cm)	Age class‐sex
Pa‐01	23.5	8.0	31.5	Juvenile male	Pa‐05	32.5	12.0	44.5	Adult‐Male
Pa‐02	36.5	13.5	50.0	Adult female	Pa‐06	33.0	13.0	46.0	Adult‐Female
Pa‐03	35.0	12.1	47.1	Adult male	Pa‐07	34.0	13.9	46.0	Adult‐Female
Pa‐04	36.0	14.0	50.0	Subadult male	Pa‐08	32.0	12.0	44.0	Subadult‐Male

	E‐L right (cm)	E‐L left (cm)	HL‐L right (cm)	HL‐L left (cm)		E‐L right (cm)	E‐L left (cm)	HL‐L right (cm)	HL‐L left (cm)
Pa‐01	1.3	1.3	5.1	5.2	Pa‐05	1.6	1.8	7.1	7.1
Pa‐02	2.1	2.1	7.1	6.9	Pa‐06	2.0	1.7	7.3	7.0
Pa‐03	2.0	2.0	7.2	7.2	Pa‐07	1.8	1.7	7.2	7.2
Pa‐04	1.7	1.7	7.4	7.2	Pa‐08	0.7	0.7	6.9	6.9

Abbreviations: E‐L, right and left ear length; H‐BL, head‐body length; HL‐L, hind leg length; T‐L, tail length; Total L, total length.

Male penis length (P‐L) ranged from 1.1 to 1.6 cm, while anus–penis distance (AP‐D) for adult males was on average 4.2 cm (Table 3). For females, anus–vulva distance (AV‐D) was between 0.5 and 1.2 cm, and clitoris length (C‐L) was from 1.2 to 2.0 cm (Table 3).

**TABLE 3:** Morphometrics on reproductive traits for eight *Plagiodontia aedium*.

ID	Sex	Age class	AP‐D (cm)	P‐BL (cm)	P‐L (cm)	AV‐D (cm)	V‐BL (cm)	C‐BL (cm)	C‐L (cm)
Pa‐01	Male	Juvenile	1.1	0.2	1.1				
Pa‐02	Female	Adult				1.2	0.7	0.4	1.2
Pa‐03	Male	Adult	4.3	1.1	2.6				
Pa‐04	Male	Subadult	3.8	1.0	2.4				
Pa‐05	Male	Adult	4.1	1.0	2.1				
Pa‐06	Female	Adult				0.6	nd	0.9	2.0
Pa‐07	Female	Adult				0.5	0.6	0.5	1.4
Pa‐08	Male	Subadult	2.5	0.7	1.7				

Abbreviations: AP‐D, anus‐penis distance; AV‐D, anus‐vulva distance; C‐BL, clit base length; C‐L, clitoris length; nd, no data; P‐BL, penis base length; P‐L, penis length; V‐BL, vulva base length.

Table 4 presents a framework of weight and physiological reference values from Carpenter and Marion (2018) for several rodent species. Data corresponding to *P. aedium*, displayed in Table 4, were taken from three adult females and five males, of which, three were adults, one was a subadult, and the remaining were juveniles (Table 2).

**TABLE 4:** Weight and physiological comparison between *Plagiodontia* aedium and other rodents according to Carpenter and Marion (2018).

Species	Average weight (kg) (male/female)	Temperature (°C)	Cardiac frequency (beats/min)	Respiratory frequency (breaths/min)
*Plagiodontia aedium*	0.4–1.4/ 1.3–1.6	35.2–37.9	168–332	76–128
*Cavia porcellus*	0.9–1.5/0.7–1.0	37.5–39.5	230–380	40–120
*Chinchilla lanigera*	0.45–0.6/0.55–0.8	34.9–37.9	200–240	40–80
*Cynomys ludovicianus*	1.0–2.2/0.5–1.5	35.4–39.1	150–320	30–60
*Rattus norvegicus*	0.35–0.5/0.25–0.35	37.5–39.5	250–450	70–120

### 3.4 Clinical Profile

The cardiac and respiratory frequencies (CF and RF) were 227 and 95.5, respectively, while the body temperature (T) was 36.9°C on average (Tables 2 and 5). Cardiac frequency (CF) and respiratory frequency (RF) vary slightly when measured post‐capture and pre‐release, where we can see some increments and decrements regardless of sex and age class across all individuals. Cardiac frequency varied between 168 and 332 beats per minute (B/min), and respiratory frequency was observed in a range of 44–156 respirations per minute (R/min) (Table 5).

**TABLE 5:** Physiological constants of hutia specimens included in this study.

Family	ID	Post‐capture		
		CF (B/min)	RF (R/min)	*T* (°C)
1	Pa‐01	320	128	36.2
	Pa‐02	332	80	37.4
	Pa‐03	192	112	36.6
	Pa‐04	212	96	37.5
2	Pa‐05	240	88	36.7
	Pa‐06	176	84	35.2
	Pa‐07	176	100	37.9
	Pa‐08	168	76	37.9

Family	ID	Pre‐release		
		CF (B/min)	RF (R/min)	*T* (°C)
1	Pa‐01	248	156	na
	Pa‐02	228	80	na
	Pa‐03	188	68	na
	Pa‐04	200	44	na
2	Pa‐05	224	56	36.0
	Pa‐06	228	88	36.6
	Pa‐07	188	112	35.4
	Pa‐08	204	104	35.8

Abbreviations: B/min, beats per minute; CF, cardiac frequency; RF, respiratory frequency; *T*, rectal temperature in°C.

Only two individuals, one juvenile and two subadults, showed pathologies in the eyes or oral cavity. For the skin and its annexes, in Pa‐03, an alopecic area was observed in the hindquarters, dorso‐caudal region, as well as a scar on the left paw, latero‐lateral plantar region. However, in Pa‐07, secretion and slight vulvar dilation were observed. In four of the eight specimens, mites from the hindquarters were collected, both in the ventral and dorsal regions of the individual.

Sexing of the specimens was carried out with the naked eye. Females have a hypertrophied clitoris like a penis; this structure is cranial to the vulva, and the anal orifice is caudal to the vulva. In males, the penis is located cranial to the anal orifice, with an average distance of 3.16 cm (mean of the distance between the anus and the penis, AP‐D, for males is included in this study) (Table 3).

For phlebotomies, venipuncture was attempted in the saphenous vein, managing to collect 3 mL of whole blood from the Pa‐04 specimen. This volume was distributed in two collection tubes, one with EDTA and the other without additives, to study the hematic and biochemical profiles. Results for the 25 tests in addition to the blood profile are shown in Table S4. No out‐of‐range values were observed based on the rodent parameters used in National Bio Vet Laboratory (NBVL).

Stool analysis for the three individuals was sent to National Bio Vet Lab located in Florida, United States, Pa‐01, Pa‐02, and Pa‐04. For all stool samples analyzed, no eggs, larvae, or adult forms of parasites were observed.

Most of the results of the hemogram and blood chemistry performed on the *P. aedium* specimen coincide with the values established for other rodents (Table 6). When the value of *P. aedium* is compared to four other rodent species, 16 out of 35 parameters (leukocytes, hemoglobin, hematocrit, monocytes, basophils, sodium, chloride, BUN, creatinine, total protein, albumin, globulin, calcium, bilirubin total, and triglycerides) fell within the range established for the other four species included in Table 6. Other parameters show a clear difference from other rodents included in Table 6, such as neutrophils, lymphocytes, phosphorus, and ALP.

**TABLE 6:** Comparison of hematologic and serum biochemical values between *Plagiodontia aedium* and other rodents according to Carpenter and Marion (2018)^1^.

Meas	*Plagiodontia aedium*	^1^ *Cavia porcellus*	^1^ *Chinchilla lanigera*	^1^ *Cynomys ludovicianus*	^1^ *Rattus norvegicus*	Units
Leukocytes WBC	7.7	7–14	5.4–15.6	1.9–10.1	5–23	10 × 3/μL
Erythrocytes RBC	4.27	4–7	5.6–8.4	5.9–9.4	7–10	10 × 6/μL
Hemoglobin Hgb	13.3	11–17	11.8–14.6	12.7–19.6	12–18	g/dL
Hematocrit PCV	43.8	35–45	27–54	36–54	35–45	%
Neutrophils	80	20–60	39–54	43–87	10–50	%
Lymphocytes	12	30–80	45–60	8–54	50–70	%
Monocytes	1	2–20	0–5	0–12	0–10	%
Eosinophils	6	0–5	0–5	0–10	0–5	%
Basophils	1	0–1	0–1	0–2	0–1	%
Glucose	149	60–125	109–193	120–209	50–135	mg/dL
Sodium	135	146–152	142–166	144–175	135–155	mmol/L
Potassium	7.2	6.8–8.9	3.3–5.7	4–5.7	5.9	mmol/L
Chloride	97	98–115	108–129			mmol/L
BUN	17.8	9–32	17–45	21–44	15–21	mg/dL
Creatinine	1.2	0.6–2.2	0.4–1.3	0.8–2.3	0.2–0.8	mg/dL
Total protein	7	4.6–6.2	3.8–5.6	5.8–8.1	5.6–7.6	g/dL
Albumin	3.7	2.1–3.9	2.3–4.1	2.4–3.9	3.8–4.8	g/dL
Globulin	3.3	1.7–2.6	0.9–2.2	3.4–4.2	1.8–3	g/dL
Calcium	11	7.8–10.5	5.6–12.1	8.3–10.8	5.3–13	mg/dL
Phosphorus	3.2	5.3	4–8	3.6–10	5.8–8.2	mg/dL
Bilirubin, total	0.34	0.3–0.9	0.6–1.3	0.1–0.3	0.2–0.6	mg/dL
ALP	177		6–72	25–64	16–96	U/L
ALT (SGPT)	44	10–25	10–35	26–91	20–92	U/L
AST (SGOT)	49		96	16–53		U/L
Cholesterol	136	20–43	50–302		40–130	mg/dL
Triglycerides	138	0–145			26–145	mg/dL

Abbreviation: Meas, measurements.

The test result for alkaline phosphatase (ALP) of *P. aedium*, in contrast to the values of *Chinchilla lanigera*, *Cynomys ludovicianus* and *Rattus norvegicus*, is elevated. However, in the post‐capture and pre‐release clinical evaluations of the sampled specimen, no jaundice or any other sign that could suggest liver disease was observed.

### 3.5 Food Preference

Both families remained approximately 15 days in holding pens, and during this time, a total of 15 plant species were offered, of which five came from the local market and ten from their natural habitat (Table 7).

**TABLE 7:** Offered and consumed plant species during quarantine.

	Offered food items during quarantine			Consumed food items	
	Species name	Common name (Spanish)	Plant part	Family 1	Family 2
Natural Habitat	*Clusia rosea*	Copey	Leaves	X	X
	*Guarea guidonia*	Cabirma	Leaves	X	X
	*Eugenia foetida*	Escobon	Leaves	X	
	*Hura crepitans*	Jabilla	Leaves		
	*Mangifera indica*	Mango	Leaves		X
	*Chrysophyllum cainito*	Caimito	Leaves		X
	*Mastichodendron foetidissimun*	Caya amarilla	Leaves		
	*Guazuma ulmifolia*	Guacima	Leaves		X
	*Gouania polygama*	Bejuco de hutía, bejuco de indio	Branch	X	X
	*Trichostigma octandrum*	Pabellón	Leaves	X	
Local Market	*Daucus carota*	Zanahoria	Root	X	X
	*Zea maiz*	Maíz	Fruit and leaves		
	*Musa paradisiaca*	Plátano	Fruit		
	*Ipomea batatas*	Batata	Root		
	*Xanthosoma sagittifolium*	Yautía blanca	Root		

#### 3.5.1 Family 1

This family consumed a total of six different plant species during their acclimation period within the enclosure. Food items from the natural habitat, specifically leaves, were the most preferred (83%), while supplementary food items being less preferred (17%). The two most consumed plant species were *Clusia rosea* (Copey) and *Guarea guidonia* (Cabirma). The least consumed species from their habitat were *Gouania polygama* (Bejuco de hutia) and *Trichostigma octandrum* (Pabellón). From the supplementary food items given, the individuals showed interest only in *Daucus carota* (carrot).

#### 3.5.2 Family 2

This family consumed a total of seven different plant species during the acclimation period. Food items from the natural habitat, specifically leaves and branches, were the most preferred (85%), while supplementary food items being less preferred (14%). The two most consumed plant species were *C. rosea* (Copey) and *G. guidonia* (Cabirma). The least consumed species from their habitat was *E. foetida* (Escobon). From the supplementary food items given, the individuals showed interest only for *D. carota* (carrot).

Six out of fifteen plant species offered were not consumed by either family of hutia. Four out of the 15 plant species offered were consumed by both families (Table 7).

### 3.6 Behavior During Manipulation and the Acclimation Period

The four individuals from Family 1 remained alert during the clinical evaluation, while the individuals from Family 2 were found to be docile and calm, except for specimen Pa‐05, who maintained an alert and aggressive behavior throughout the procedure.

The videos from camera traps located in the enclosure revealed that the specimen Pa‐05, belonging to Family 2, crossed to the side of the quarantine area occupied by Family 1 and fought with the alpha male identified as Pa‐03. From this fight, Pa‐03 was left with a shallow scratch wound, close to the nose, while Pa‐05 received three bite wounds, one on the right front paw and two on the tail. The day of the fight coincided with the release of Family 1. Prior to the transfer of this family to the release area, specimen Pa‐03 had its wound cleaned and disinfected with potable water and chlorhexidine soap. Subsequently, an antibiotic cream based on sulfonamide, lidocaine, and vitamin A was applied. The same treatment was given to specimen Pa‐05 once a day for 6 days, until the release of Family 2, with the difference that after each cure a sterile gauze bandage was applied.

At the time of release of specimen Pa‐05, the wound on the right front paw was seen to have healed almost completely, and in the videos of the quarantine, it was observed that the prehensile capacity of the tail was not affected.

### 3.7 Monitoring Post‐Release

Monitoring of both families took place from June 1 until August 31 of 2022. Data from two camera traps per burrow were compiled, and the behavior of the individuals was classified into 15 different observed behaviors. Relative frequency of the behaviors was estimated based on the total amount of times the behavior occurred. Only one family remained in the burrows selected for translocation. The other family abandoned theirs during the first week after release.

#### 3.7.1 Family 1

Four members of Family 1 were released in PB‐05 on June 1 of 2022. After the release, the first individual sighting was at 11:14 PM on June 4. The female with her offspring was observed the same day at 11:27 PM. The temperature range during first sightings oscillated between 25°C and 26°C. The last recorded sighting of hutia in PB‐05 was on August 23, between 6 and 8 AM. The activity hours for this family were from 7:37 PM to 7:43 AM, with temperatures ranging from 21°C to 27°C. The maximum number of animals observed simultaneously in cameras was three.

Eleven different types of behaviors were observed for Family 1 (Table 8). Behavior frequency was recorded per individual per video, gathering a total of 103 entries for Family 1. Common behaviors for the group such as grooming and socializing were observed using Reconyx camera traps which record 20 s videos at a time. The two most common behaviors observed were burrow entry (30%) and animals passing by the entrance (17%). Burrow exit (14%) was observed in lesser frequency, suggesting the presence of an additional exit not found in this study. Several behaviors supporting the optimal quality of the burrow were observed, such as the introduction of vegetation (6%) to the burrow and foraging near the entrance (9%). Vocalizations occurred between 10 and 11 PM, with temperature ranging from 21°C to 24°C.

**TABLE 8:** Frequency of behaviors observed during monitoring of Family 1 in Mejita.

Behavior	Frequency	Relative frequency	Time ranges
Social grooming	1	0.01	7:37 PM
Alert status	1	0.01	8:39 PM
Running	3	0.03	9:47 PM–10:47 PM
Burrow entry	31	0.30	7:37 PM–5:58 AM
Vegetation entry	6	0.06	5:13 AM–5:52 AM
Exploration	5	0.05	7:37 PM–4:32 AM
Passing by	17	0.17	7:49 PM–4:38 AM
Foraging	9	0.09	1:25 AM–5:18 AM
Sniffing	14	0.14	7:48 PM–7:43 AM
Burrow exit	14	0.14	7:56 PM–6:03 AM
Vocalization	2	0.02	10:33 PM–10:44 PM
	103	1.00	

Two invasive species were detected during monitoring, rats and one feral cat. Rats occupy the burrow, coming in and out during nocturnal hours, suggesting that they reside in the burrow along the hutias. One feral cat was detected passing by on July 1 at 11:29 PM.

#### 3.7.2 Family 2

Four members of Family 2 were released in PB‐04 on June 7 of 2022. After the release, the first individual sighting was at 10:10 AM on the same day of the release. Temperature during first sighting was 26°C. The last recorded sighting for this family in PB‐04 was on June 14 at 12:43 AM with an ambient temperature of 22°C. Temperature during hutia outings oscillated between 22°C and 35°C. Individuals from this family had a wide range of activity hours, 12:24 PM until 11:33 PM. Individuals came out of the burrow during morning and afternoon hours.

Thirteen different behaviors were observed (Table 9). A total of 104 observations were quantified and its relative frequency calculated. The two most common behaviors observed were burrow exit (29%) and burrow entry (19%), and third, animals passing by (13%). Individuals from this family were walking from one side to the other repeatedly during their first day. One of the individuals was seen scratching multiple times, and playful behavior between individuals of the same family was also observed.

**TABLE 9:** Frequency of behaviors observed during monitoring of Family 2 in Mejita.

Behavior	Frequency	Relative frequency	Time ranges
Individual recognition	1	0.01	2:37 AM
Running	4	0.04	7:49 PM–9:58 PM
Burrow entry	20	0.19	8:02 PM–4:03 AM
Exploration	4	0.04	7:28 PM–9:17 PM
Passing by	14	0.13	7:32 PM–2:24 AM
Foraging	2	0.02	7:22 PM–7:25 PM
Sniffing	10	0.10	5:42 PM–1:48 AM
Burrow exit	30	0.29	5:42 PM–11:05 AM
Vocalization	8	0.08	8:38 PM–1:55 AM
Teeth filing	4	0.04	7:22 PM–2:28 AM
Playing	3	0.03	9:09 PM–1:07 AM
Defecating	1	0.01	7:31 PM
Scratching	3	0.03	7:29 PM–3:15 AM
	104	1.00	

Two invasive species were detected during monitoring: rats and mongoose of different age classes. Rats shared residency with the family within the burrow, while mongoose was observed at a higher frequency passing by, entering, and exploring the area of the burrow frequently.

## 4 Discussion

### 4.1 Sampling

Little is known about the family structure and dynamics of Hispaniolan hutia. The family group structures observed for the families captured in San Juan Hill were very heterogeneous, with the presence of male and females of different ages. In both families, there was the presence of one adult male that could be considered dominant, as shown by their aggressive interaction during the acclimation period. It has been seen that in Cuban hutias, adults tolerate subadult males and can be considered the part of the group that eventually disperses [Comas et al. 1994 cited by Silva Taboada, Duque, and Franco (2007)]. Similar social group patterns have been seen in other rodents, where there is a single dominant adult male, with several females and subadult or juvenile males in the family group (Escobar and Cecilia 2012; Romero, Legorreta, and ICD 2005).

### 4.2 Habitat Characterization and Selection

Most studies on hutias habitat characterization focuses on the description of the environment they inhabit, with special emphasis on limestone rocky areas and the vegetation it presents (Kennerley, Nicoll, Butler, et al. 2019; Kennerley, Nicoll, Young, et al. 2019; Turvey et al. 2020); however, very little is known about the plant species that hutia uses in its environment. The vegetation use determined though transects executed in the proximities of active burrows in San Juan Hill allowed us to better understand how these animals use their surrounding habitat. Due to the low dispersal range reported for hutias (Kennerley, Nicoll, Butler, et al. 2019), the key aspects of habitat quality are the availability of unoccupied burrows and the associated vegetation, both for food and teeth filling (Kennerley, Nicoll, Young, et al. 2019).

The presence of key plant species in hutia habitat is crucial for the successful establishment of the families, since these provide food and shelter. *Clusia* sp., *Piper* sp., *Guasuma* sp., and *Eugenia* sp. (Borroto Páez et al. 2011; Martínez, Linares, and Ramírez 2017; Martínez, Rodríguez, and Sospedra 2005; Silva Taboada, Duque, and Franco 2007) are some of the genera present in the Caribbean islands that are commonly consumed by different endemic rodents, including those in this study (Table S3). Mejita habitat has a wide complexity and habitat heterogeneity, conditions that have been reported as necessary for the establishment of small tropical mammals (August 1983). *Cupania americana*, *Guasuma tomentosa* from the low‐level vegetation, *Piper aduncum*, and *C. cainito* (shrub stage) in the mesoforest are all species present in Mejita which provide a denser undergrowth forest increasing canopy closure and higher tree cover. These latter habitat characteristics have been reported by Kennerley, Nicoll, Young, et al. (2019) as key parameter predictors that indicate a high probability of use by the species.

The criteria determined for the evaluation of habitat quality must be taken with caution, given that the criteria incorporated in the ranking matrix included data extracted from a wide range of habitats taken by Kennerley, Nicoll, Young, et al. (2019) across the Dominican Republic. Also, this area is highly impacted by mining since 1975; therefore, habitat quality criteria established in this study should take into consideration that the individuals that have survived are occupying some of the last available habitats and might have adapted to higher levels of impact (noise, human presence, feral cats nearby, and others), and data from other populations should be included to establish more general parameters.

### 4.3 Animal Processing

Based on the morphometric data obtained from these eight individuals, subtle size differences between males and females are observed when we considered body weight and body length; however, when reproductive structures are measured, the distance between vulva/penis and anus is clearly different between the sexes. Observations regarding their reproductive external organs indicate that specimens from both sexes share a structure that at first glance is very similar to a penis, but in females, it appears to be a hypertrophied or elongated clitoris. This elongated clitoris has been described for the Cuban hutia Conga with a length of 25 mm occluding the vaginal opening (Angulo 1945). Although females have a hypertrophied clitoris like a penis, this structure is cranial to the vulva, and the anal orifice is caudal to the vulva, observing these three contiguous structures in the female. While in males, the penis is located cranial to the anal orifice, at an average distance of 3.18 cm (mean of the distance registered). In addition, a big difference between the distance of anus and vulva and the anus and penis, of females and males, respectively, was observed (Table 3). However, the determined traits that show clear sexual dimorphism are hard to determine through observation alone, and females can frequently be mistaken by males if the animals are not handled and sexual structures observed by experienced observers in the field (Angulo 1945), increasing the risk of stress to these mammals.

Regarding the length of the penis and the diameter of the base of the penis, for the Pa‐03 specimen, it was 2.6 and 1.1 cm, respectively, while the length of the penis of the Pa‐05 specimen was 2.1 and 1 cm in the diameter of its base (Tables 2 and 3). If we compare these values with those obtained by Angulo and Alvarez (1948), where the length of the penis of adult males *C. pilorides* under normal conditions was between 4 and 6 cm and its diameter was 1 cm, it is inferred that *C. pilorides* doubles and almost triples the length of the penis of *P. aedium*, while its diameter is the same for both species. Suggesting clear anatomical differences between the species, differences are expected due to the much bigger size of *C. pilorides*.

Morphometric data also suggest a decrease in weight of the animals (Table 2) and a tendency where the higher the number of days spent in the corral the larger the weight loss. Weight loss may have been caused by multiple factors, such as insufficient food was being provided for the number of animals within the enclosure; also, hierarchical rivalries may have been affecting the feeding time of females, juveniles, and subadult males. For example, in Table 2 for Family 1, the largest weight difference was observed in the only female present in the family, and for Family 2, the largest weight difference was also for an adult female. On the other hand, subadult males for both families presented the least amount of weight loss, suggesting they were not affected by the dominant male territorial dominance. These subadult males were also found to cross the mesh that divided both families to forage in the contiguous territory within the enclosure. Finally, it is important to notice that these behaviors are being reported for only eight individuals, and a bigger sampling size needs to be studied to better understand their foraging behavior in captivity.

The decrease in weight observed (Table 2) may have been biased by multiple factors, such as the lack of experience from the team, where food items offered were initially selected based on the plant list obtained from their natural habitat and later modified based on our daily observations of what plant species were most preferred by the animals. There is no information about the species' dietary needs. Also, wild animals need time to acclimate to a captive environment, and every individual presents different levels of stress during handling, some showing to be more aggressive than others, potentially inserting some bias during weight measurement.

Due to the limited existing literature regarding morphometry, physiological constants, and hematic and biochemical values for *P. aedium*, multiple rodent species, such as the Cuban hutia (*C. pilorides*), Chinchilla (*C. lanigera*), the Prairie Dog (*C. ludovicianus*), and the rat (*R. norvegicus*), are taken as reference for comparative values. Fuente Arzola (2019) studied the morphometric variations of 768 adult individuals of *C. pilorides* considering absolute values of measurements such as: body weight, total length, head–trunk length, tail length, and legs (including the nail). Similarly, Borroto Páez (2002) examined 556 specimens of 11 species of hutias and established that the most representative measurements of the size of a species are: body weight, body length, and total length, the latter being dependent on the length of the tail. It was also determined that the average weight of adult Capromyds varies between 3.8 kg for C. pilorides and 0.48 kg for *Mesocapromys angelcabrerai* and that the weights of the species of the genera *Mysateles* fall within these intervals, with species that average between 1.23 and 1.80 kg, *Geocapromys* genus with an interval between 0.74 and 1.74 kg, and *Plagiodontia* genus with an average of 0.94 kg. The two male adults of *P. aedium* in the present study weighed 1.4 kg (Tables 2 and 3), exceeding the average value established for specimens of the genus *Plagiodontia*. Taking other species into account, the 16 adult male specimens of *C. pilorides* that Angulo and Alvarez (1948) worked with had an average weight between 3 and 5 kg. Based on these values, it can be inferred that *C. pilorides* doubles and can even quintuple the weight of *P. aedium*.

Echevarría and Miazzo (2002) state that temperature, wind, humidity, and rain reduce the thermal insulation of the hair layer, increasing the loss of convective heat and decreasing the effective temperature. Hence, temperature may fluctuate depending on environmental conditions during the time of capture and processing (Table 5). When the physiological parameters of *P. aedium* are compared to four other rodent species in Table 4, the body temperature for *P. aedium* was almost like that of *C. lanigera* despite the average weight for male and female *C. lanigera* being smaller than that of *P. aedium*. The other rodent species in Table 4 presented a higher upper range body temperature, reaching up to 39.5°C. Body temperature measurement during specimen capture may be dependent on the environmental factors described by Echevarría and Miazzo (2002) and cannot be taken as absolute values due to the influence of external ambient temperature.

Cardiac frequency of *P. aedium*, when compared to four other rodent species included in Table 4, was more like that of *C. ludovicianus*, the black‐tailed prairie dog, while respiratory frequency was more like that of the brown rat, *R. norvegicus*. Hence, *P. aedium* needs to be evaluated at a population level, and more information regarding the species physiology needs to be taken to better determine the species range parameters.

### 4.4 Clinical Profile

Table 5 shows the results from physiological parameters taken from the animals. Cardiac and respiratory frequencies reported for eight specimens were variable post‐capture and pre‐release, some individuals being calmer than others during handling. Cardiac frequency was as high as 332 B/min, and respiratory frequency 156 R/min. These values stand in the lower rates when compared with rats and mice, where rats can increase their cardiac frequency up to 350–370 B/min (Bolter and Atkinson 1988; Lujan et al. 2012) and mice up to 500–800 B/min (Fewell et al. 1997; Lujan et al. 2012). On the other hand, the biochemical profile shown in Table S5 can serve as a baseline for future studies. Although the results of all the tests are within the normal parameters of a rodent, there are no reference values for the species; hence, these must be constructed with more individuals from different populations.

The differences in blood results for a species can be influenced by multiple factors, as evidenced in the work of Soriano Soriano (2015) where the blood profile of 30 specimens of *C. porcellus* was performed, with samples obtained by cardiac puncture, determining a glucose value of 84.2 mg/dL, protein of 4.41 g/dL, and total cholesterol of 46.7 mg/dL, concluding that the results of their work differed with those reported by other authors because these values are dependent on the type of food and the rations consumed by the animals handled.

The implications of the results of the hematic and biochemical values of *P. aedium* obtained in the present study will serve as a starting point to continue research that will allow establishing reference values for the species; since, currently, there is no reference literature and the comparison of parameters, considering those established for other species of hutias and other rodents leads to a wide degree of uncertainty. Increasing the number of individuals and adding more populations are crucial to better understand the physiological parameters for the species, promoting better care for hutias in the future.

### 4.5 Food Preference

Plant species preferred by *P. aedium* were *C. rosea* (Copey) and *Guarea guidonia* (Cabirma), both native to Hispaniola. These are tall woody plants commonly associated to their habitat. Similar trophic habits were found in the hutia conga *C. pilorides* from Cuba, 3 times heavier than the Hispaniolan hutia (3782 g), (Borroto Páez et al. 2011), which has also been found to consume Copey and Cabirma in Cuba [Comas et al., 1993 cited by Silva Taboada, Duque, and Franco (2007)].

The only supplementary food consumed by hutias was carrots. In a study published by Johnson, Taylor, and Winnick (1975) on Capromys rodent at Tacoma Zoo, they found that the preferred food items by *P. aedium* in captivity were yams, sweet potatoes, and carrots. Thus, the species can be maintained in captivity with these supplemental food items when natural plant species from their habitat is not available. However, during captivity, they clearly preferred native plant species over supplemental diet. This also may have been driven by the fact these individuals were not raised in captivity and were only adapted to eat native vegetation from the wild.

Further studies regarding the use *P. aedium* gives to native plant species need to be conducted to determine which parts of the plants are being consumed for feeding purposes and which are being gnawed for teeth‐filling purposes.

### 4.6 Behavior

During the acclimation period, two dominant males fought within the corral. Similar behavior was observed by Johnson, Taylor, and Winnick (1975), in which individuals in captivity were observed fighting, one fatally wounding the opponent. In captivity, individuals are more likely to show hierarchical rivalries, given the limitation of space and foraging locations within the enclosure (Silva Taboada, Duque, and Franco 2007). This suggests the need to have bigger enclosures with additional foraging sites within the enclosure based on the number of males present, to prevent hierarchical rivalries. It should be noted that the separation between both families was a metal mesh of few centimeters, with holes, so both families had visual and tactile contact through the mesh, which could have increased the aggressiveness of the males. Individual recognition by sniffing through the mesh was observed during monitoring videos in quarantine. Therefore, better isolation of different families is recommended for future translocation activities.

### 4.7 Translocation as a Conservation Measure

Translocation has been widely used to ensure the conservation of species (Cade, Burnham, and Burnham 2003; Griffith et al. 1989; Knapp and Hudson 2005; Miskelly and Powlesland 2013; Short 2009); however, translocation programs are highly dependent on appropriate habitat selection and on having extensive knowledge of the species requirements. When rare and understudied species are targeted for translocation programs, there is a higher risk of failure. This translocation exercise was fully successful for one of the two families. The survival rate expected for Family 1, given that during the monitoring post‐release, two behaviors were observed which suggested establishment of Family 1 in PB‐05, introduction of vegetation to the burrow (6%) and foraging near the entrance (9%) (Table 8). Neither of these behaviors were observed at a high rate in Family 2 (Table 9). However, the early abandonment of the selected burrow from Family 2 suggests the inadequacy of the burrow for residency and the quick migration of the family to another location. Longer monitoring studies on translocation sites should be included in the future to evaluate the long‐term survival of both families and properly assess the success of the translocation programs.

Finally, these data suggest that translocation programs for *P. aedium* can have a high success rate as long as an appropriate habitat characterization is performed. The more information is gathered, the higher is the likelihood of a successful translocation. Given the lack of data on local populations, we recommend additional population studies of *P. aedium* on Hispaniona, thus allowing for better management decisions and survivability of the species in the long term.

## Author Contributions

**Miguel S. Núñez‐Novas:** conceptualization (equal), data curation (equal), formal analysis (equal), funding acquisition (lead), investigation (lead), methodology (equal), writing – original draft (equal), writing – review and editing (equal). **Rosanna Carreras‐De León:** conceptualization (equal), data curation (equal), methodology (lead), writing – original draft (equal), writing – review and editing (equal). **Amelia L. Mateo Jiménez:** conceptualization (equal), data curation (equal), investigation (equal). **Carolina Dávila:** investigation (equal), methodology (equal), writing – original draft (equal), writing – review and editing (equal). **Pilar Calderón:** data curation (equal), project administration (equal), writing – review and editing (equal).

## Conflicts of Interest

Natalus Consultoría Ambiental S.R.L. was hired by *Barrick Pueblo Viejo Dominicana Jersey 2 Limited* to execute this study. Pilar Calderón was the Manager of the Environmental Department from *Barrick Pueblo Viejo Dominicana Jersey 2 Limited* during the execution of this study.

## Supporting information


**Table S1.** Criteria for classifying the quality of a hutia burrow.
**Table S2.** List of 24 plant species associated to active burrows in the study area.
**Table S3.** Presence–absence of plant species in the area of occupation of active and potential burrows included in this study.
**Table S4.** Plant species list identified in the eight transects done in San Juan Hill and Mejita sites.
**Table S5.** Left: Results of the biochemical profile (chemistry) of the specimen Pa‐04. Right: Results of the blood profile (hematology) of the specimen Pa‐04.

## Data Availability

Upon publication of the manuscript, the authors will make all data publicly available on Dryad. To access the private repository for peer review please follow the link: https://datadryad.org/stash/share/T__‐nu_Tx7y2UYuTNGNr3sl1wnHMb‐F6dH8cmpvB484. DOI:10.5061/dryad.dr7sqvb5z.
