# Correction to “Rescue and Translocation of Hispaniola Hutia (*Plagiodontia aedium*) in Pueblo Viejo, Cotuí Mining Concession Area”

**DOI:** 10.1002/ece3.73715

**Published:** 2026-06-02

Núñez‐Novas, M.S., Carreras‐De León, R., Mateo Jiménez, A.L., Dávila, C. and Calderón, P. (2024), Rescue and Translocation of Hispaniola Hutia (Plagiodontia aedium) in Pueblo Viejo, Cotuí Mining Concession Area. Ecol Evol, 14: e70560. https://doi.org/10.1002/ece3.70560.

An additional affiliation has been added for Miguel S. Núñez‐Novas, as follows:

Instituto Geográfico Universitario, Universidad Autónoma de Santo Domingo, Zona Colonial, Santo Domingo, Dominican Republic.

The online version of this article has been corrected accordingly.

We apologize for this error.

