# Supplementary material for: Rescue and Translocation of Hispaniola Hutia (Plagiodontia aedium) in Pueblo Viejo, Cotuí Mining Concession Area
Source: Ecol Evol. 2024 Nov 13;14(11):e70560. doi: 10.1002/ece3.70560 (PMC11560802; doi:10.1002/ece3.70560)
Supplement: Supplementary file 1 — Table S1. Criteria for classifying the quality of a hutia burrow. Table S2. List of 24 plant species associated to active burrows in the study area. Table S3. Presence–absence of plant species in the area of occupation of active and potential burrows included in this study. Table S4. Plant species list identified in the eight transects done in San Juan Hill and Mejita sites. Table S5. Left: Results of the biochemical profile (chemistry) of the specimen Pa‐04. Right: Results of the blood profile (hematology) of the specimen Pa‐04. [file ECE3-14-e70560-s001.docx]

Supplemental Table 1. Criteria for classifying the quality of a hutia burrow.

| Criteria | Scoring in Points** | | | **Final Score |
| --- | --- | --- | --- | --- |
|  | **1** | **2** | **3** |  |
| 1. Altitude | Altitude ≤ 120 masl | Altitude ≥ 450 masl | Observed range in PV (120-449 masl) |  |
| 1. Number of entrances | No entrance | One entrance | Multiple entrances |  |
| 1. Slope | ≤ 30° | 31-45° | > 45° |  |
| 1. Soil temperature | ≥ 23.5 C | ≤ 19.9 C | 20.0-23.4 C |  |
| 1. Canopy cover over entrance | 70-79% | 80-89% | 90-100% |  |
| 1. Composition of inside walls | Tree roots and trunks | Karst rock with other substrate | Karst walls and soil with black dirt |  |
| 1. Internal tunnels and Access pathways | None | Only one tunnel and one access | More than one entry path |  |
| 1. Invasive species | Cats and dogs | Mongoose and rats | None |  |
| 1. Distance between active burrows | ≤ 34 m | 35-49 m | ≥ 50 m |  |
| 1. Associated vegetation (see Supplemental Table 1) | Less than 29% of reported species | Between 30-49% of reported species | More than 50% of reported species |  |
| TOTAL | | | |  |

Note: Dimensions and directionality of the burrows were not included.

**Scoring points are based in three levels of quality, where ´1 point´ is the lesser quality characteristics and ´3 points´ are the higher quality characteristics determined by experts on the field.

**Score interpretation =

| Burrow with optimal conditions | 25-30 points |
| --- | --- |
| Burrow with favorable conditions | 20-24 points |
| Burrow that requires alterations to improve conditions | 15-19 points |
| Unfit burrow | 10-14 points |

Supplemental Table 2. List of 24 plant species associated to active burrows in the study area.

| No. | Family | Species Name |
| --- | --- | --- |
| 1 | Anacardiaceae | *Spondias mombin* |
| 2 | Arecaceae | *Roystonea borinquena* |
| 3 | Bignoniaceae | *Spathodea campanulata* |
| 5 | Clusiaceae | *Calophyllum calaba* |
| 5 | Commeliaceae | *Commelina erecta* |
| 6 | Euphorbiaceae | *Hura crepitans* |
| 7 | Lauraceae | *Nectandra coriacea* |
| 8 |  | *Ocotea leucoxylon* |
| 9 | Melastomataceae | *Clidemia hirta* |
| 10 | Meliaceae | *Guarea Guidonia* |
| 11 |  | *Trichilia hirta* |
| 12 | Moraceae | *Ficus americana* |
| 13 | Orchidaceae | *Oeceoclades maculata* |
| 14 | Piperaceae | *Piper aduncum* |
| 15 |  | *Peperomia cf. dondoensis* |
| 16 | Primulaceae | *Theophrasta americana* |
| 17 | Pteridaceae | *Adiantum sp.* |
| 18 | Rutaceae | *Zanthoxylum martinicense* |
| 19 | Sapindaceae | *Cupania americana* |
| 20 | Sapotaceae | *Chrysophyllum cainito* |
| 21 | Simaroubaceae | *Simarouba amara* |
| 22 |  | *Burcera simaoruba* |
| 23 | Urticaceae | *Urera baccifera* |
| 24 | Vitaceae | *Cissus verticillata* |

Supplementary Table 3. Presence-absence of plant species in area of occupation of active and potential burrows included in this study.

| **Family** | **Species Name** | **Common name (Spanish)** | **Uses** | **Active Burrows** | | | | **Potential Burrows** | |
| --- | --- | --- | --- | --- | --- | --- | --- | --- | --- |
|  |  |  |  | **M7** | **M13** | **M14** | **M15** | **PB4** | **PB5** |
| Pteridaceae | *Adiantum sp.* |  |  |  |  |  |  | X | X |
| Simaroubaceae | *Burcera simaoruba* | Almacigo |  |  |  |  |  |  | X |
| Clusiaceae | *Calophyllum calaba* | Mara |  | X |  |  |  |  | X |
| Solanaceae | *Capsicum sp* | Aji montesino |  | X |  |  |  |  |  |
| Sapotaceae | *Chrysophyllum cainito* | Caimito | Gnawed | X |  |  |  | X | X |
| Vitaceae | *Cissus verticillata* | Bohuco caro |  |  |  |  |  |  | X |
| Melastomataceae | *Clidemia hirta* | Morita |  |  |  |  |  |  | X |
| Clusiaceae | *Clusia rosea* | Copey | Gnawed |  | X | X |  |  |  |
| Rubiaceae | *Coffea arabica* | Café |  | X |  |  |  |  |  |
| Costaceae | *Costus scaber* | Jengibre |  |  |  |  |  | X |  |
| Sapindaceae | *Cupania americana* | Guarano | Gnawed | X | X | X |  |  |  |
| Bignoniaceae | *Dolichandra unguis-cati* | Friega palo |  |  |  |  |  | X |  |
| Fabaceae | *Entada gigas* | Samo | Gnawed | X |  |  |  |  |  |
| Myrtaceae | *Eugenia foetida* | Escobon | Gnawed, scratches | X |  | X |  |  |  |
| Moraceae | *Ficus americana* | Higo |  | X |  |  |  | X |  |
| Rhamnaceae | *Gouania lupuloides* | Behuco de indio |  |  |  |  |  |  | X |
| Meliaceae | *Guarea guidonea* | Cabirma | Gnawed | X |  |  |  |  |  |
| Malvaceae | *Guasuma tormentosa* | Guasima | Gnawed | X |  |  |  |  |  |
| Euphorbiaceae | *Hura crepitans* | Jabilla |  | X |  |  |  | X | X |
| Lauraceae | *Nectandra coriaceae* | Cigua blanca |  |  |  |  |  | X | X |
| Lauraceae | *Nectandra hihua* | Cigua | Gnawed | X | X |  | X |  |  |
| Lauraceae | *Ocotea leucoxylon* | Cigua prieta | Gnawed | X |  |  |  | X |  |
| Orchidaceae | *Oeceoclades maculata* | Lengua de suegra, monja africana |  |  |  |  |  | X | X |
| Piperaceae | *Peperomia cf. dondoensis* |  |  | X |  |  |  | X | X |
| Piperaceae | *Piper aduncum* | Guayuyo | Gnawed | X |  |  |  | X | X |
| Arecaceae | *Roystonea borinquena* | Palma real |  | X |  |  |  | X |  |
| Bignoniaceae | *Spathodea campanulata* | Amapola |  |  |  |  |  |  | X |
| Anacardiaceae | *Spondias mombin* | Joboban | Gnawed |  |  | X |  | X | X |
| Araceae | *Syngonium podophyllum* | Tratra |  |  |  |  |  |  | X |
| Malvaceae | *Theobroma cacao* | Cacao |  | X |  |  |  |  |  |
| Primulaceae | *Theophrasta americana* | Guayaba de indio |  |  |  |  |  | X | X |
| Meliaceae | *Trichilia hirta* |  |  |  |  |  |  |  | X |
| Urticaceae | *Urera baccifera* | Pringamosa |  |  |  |  |  | X | X |
| Orchidaceae | *Vainella sp* | Orquidea |  | X |  |  |  |  |  |
| Zamiaceae | Zamia pumila | Samia |  |  | X |  |  |  |  |

Supplementary Table 4. Plan species list identify in the eight transects done in San Juan Hill and Mejita sites.

| **No.** | **Family** | **Especie** | **Presence in Transect** | **Presence in Circular Parcel** | **Hutia Tracks Presence** |
| --- | --- | --- | --- | --- | --- |
| **1** | Anacardiaceae | *Spondias mombin* | X | X | X |
| **2** |  | *Mangifera indica* | X | X | X |
| **3** | Araceae | *Xanthosoma sagittifolium* | X |  |  |
| **4** | Arecaceae | *Roystonea borinquena* | X | X |  |
| **5** |  | *Sabal domingensis* | X |  |  |
| **6** | Bignoniaceae | *Dolichandra unguis-cati* |  | X |  |
| **7** |  | *Spathodea campanulata* | X |  |  |
| **8** | Burseraceae | *Tetragastris balsamifera* |  | X | X |
| **9** | Cannabaceae | *Trema micrantha* | X | X |  |
| **10** | Clusiaceae | *Calophyllum calaba* | X |  | X |
| **11** |  | *Clusia clusioides* | X |  | X |
| **12** | Combretaceae | *Bucida buceras* | X |  |  |
| **13** | Commelinaceae | *Commelina erecta* | X |  |  |
| **14** | Costaceae | *Costus scaber* | X | X |  |
| **15** | Cyatheaceae | *Cyathea sp.* | X |  |  |
| **16** | Euphorbiaceae | *Hura crepitans* | X | X | X |
| **17** | Euphorbiaceae | *Ricinus communis* | X |  |  |
| **18** | Fabaceae | *Acacia macracantha* | X |  |  |
| **19** |  | *Entada gigas* | X |  | X |
| **20** |  | *Erythrina poeppigiana* | X |  |  |
| **21** |  | *Inga vera* | X |  | X |
| **22** |  | *Mimosa pudica* | X |  |  |
| **23** |  | *Ormosia krugii* |  | X |  |
| **24** |  | *Senna siamea* | X |  | X |
| **25** | Lauraceae | *Nectandra coriaceae* |  | X |  |
| **26** |  | *Nectandra hihua* | X |  | X |
| **27** |  | *Ocotea leucoxylon* | X | X | X |
| **28** | Loranthaceae | *Dendropemon sp* | X |  |  |
| **29** | Lycopodiaceae | *Lycopodium sp.* | X |  |  |
| **30** | Malpighiaceae | *Bunchosia glandulosa* |  | X |  |
| **31** | Malvaceae | *Ceiba pentandra* |  | X |  |
| **32** |  | *Guazuma ulmifolia* | X |  | X |
| **33** |  | *Theobroma cacao* | X |  |  |
| **34** |  | *Urena lobata* | X |  |  |
| **35** | Melastomataceae | *Clidemia hirta* | X | X |  |
| **36** | Meliaceae | *Guarea guidonia* | X | X | X |
| **37** | Moraceae | *Ficus americana* | X | X | X |
| **38** | Musaceae | *Musa AAB* | X |  |  |
| **39** |  | *Musa paradisiaca* | X |  |  |
| **40** |  | *Musa AAB* | X |  |  |
| **41** | Myrtaceae | *Eugenia foetida* | X |  | X |
| **42** | Myrtaceae | *Psidium guaiava* | X |  | X |
| **43** | Orchidaceae | *Oeceoclades maculata* |  | X |  |
| **44** |  | *Vainella sp.* | X |  |  |
| **45** | Pinaceae | *Pinus caribaea* | X |  |  |
| **46** | Piperaceae | *Peperomia cf. dondoensis* | X | X |  |
| **47** |  | *Piper aduncum* | X | X | X |
| **48** |  | *Piper hamalago* | X |  |  |
| **49** |  | *Piper sp* | X |  |  |
| **50** | Poaceae | *Bambusa* | X |  |  |
| **51** |  | *Urochloa brizantha* | X |  |  |
| **52** | Polypodiaceae | *Polypodium polypodioides* | X |  |  |
| **53** | Primulaceae | *Myrcine coreaceae* | X |  |  |
| **54** |  | *Theophrasta americana* | X | X |  |
| **55** | Pteridaceae | *Adiantum sp.* | X | X |  |
| **56** | Rhamnaceae | *Gouania lupuloides* |  | X |  |
| **57** |  | *Zanthoxylum martinicense* |  | X |  |
| **58** | Rubiaceae | *Coffea arabica* | X |  |  |
| **59** | Rutaceae | *Zanthoxylum martinicense* | X | X |  |
| **60** | Salicaceae | *Casearia arborea* |  | X | X |
| **61** | Sapindaceae | *Cupania americana* | X | X | X |
| **62** | Sapotaceae | *Chrysophyllum cainito* | X | X | X |
| **63** | Simaroubaceae | *Burcera simaoruba* | X |  |  |
| **64** | Solanaceae | *Solanun sp.* | X |  |  |
| **65** |  | *Capsicum sp.* | X |  |  |
| **66** | Urticaceae | *Cecropia schreberiana* | X | X | X |
| **67** |  | *Urera baccifera* | X | X | X |
| **68** | Verbenaceae | *Lantana sp.* | X |  |  |
| **69** | Vitaceae | *Cissus verticillata* | X | X |  |
| **70** |  | *Vitis sp.* | X |  |  |
| **71** | Zamiaceae | *Zamia debilis* | X |  |  |

Supplementary Table 5. Left: Results of the biochemical profile (chemistry) of the specimen Pa-04. Right: Results of the blood profile (hematology) of the specimen Pa-04.

| **CHEMISTRY** | | | **HEMATOLOGY** | | |
| --- | --- | --- | --- | --- | --- |
| **Test** | **Result** | **Units** | **Test** | **Result** | **Units** |
| **Glucose** | 149 | mg/dL | **Leukocytes WBC** | 7.7 | 10*3/ul |
| **Sodium** | 135 | mmol/L | **Erythrocytes RBC** | 4.27 | 10*6/ul |
| **Potassium** | 7.2 | mmol/L | **Hemoglobin** | 13.3 | g/dL |
| **Chloride** | 97 | mmol/L | **Hematocrit** | 43.8 | % |
| **CO_2_** | 13 | mmol/L | **MCV** | 103 | um3 |
| **Sodium/Potassium** | 19 | Ratio | **MCH** | 31.2 | pg |
| **Anion Gap** | 32 |  | **MCHC** | 30.4 | g/dL |
| **Serum Osmolality** | 288 | mosmo/kg | **Seg Neutrophils** | 80 | % |
| **Urea Nitrogen** | 17.8 | mg/dL | **Band Neutrophils** | 0 | % |
| **Creatinine** | 1.2 | mg/dL | **Lymphocytes** | 12 | % |
| **BUN/Creatinine** | 15 | Ratio | **Monocytes** | 1 | % |
| **Total Protein** | 7 | g/dL | **Eosinophils** | 6 | % |
| **Albumin** | 3.7 | g/dL | **Basophils** | 1 | % |
| **Globulin** | 3.3 | g/dL | **Seg Neutrophils** | 6.16 | 10*3/ul |
| **A/G Ratio** | 1.1 | Ratio | **Band Neutrophils** | 0 | 10*3/ul |
| **Calcium** | 11 | mg/dL | **Lymphocytes** | 0.924 | 10*3/ul |
| **Phosphorus** | 3.2 | mg/dL | **Monocytes** | 0.077 | 10*3/ul |
| **Cl/P Ratio** | 30 | Ratio | **Eosinophils** | 0.462 | 10*3/ul |
| **Bilirubin, Total** | 0.34 | mg/dL | **Basophils** | 0.077 | 10*3/ul |
| **Bilirubin Direct** | 0.14 | mg/dL | **Platelet Count** | 391 | 10*3/ul |
| **Bilirubin Indirect** | 0.2 | mg/dL |  |  |  |
| **Alk Phosphatase** | 177 | U/L |  |  |  |
| **ALT (SGPT)** | 44 | U/L |  |  |  |
| **AST (SGOT)** | 49 | U/L |  |  |  |
| **GGT** | 8 | U/L |  |  |  |
| **Creatine Kinase** | 120 | U/L |  |  |  |
| **Cholesterol** | 136 | mg/dL |  |  |  |
| **Triglycerides** | 138 | mg/dL |  |  |  |
| **Amylase** | 703 | U/L |  |  |  |
